# Chitosan Nanoparticles as a Promising Nanomaterial for Encapsulation of Pomegranate (*Punica granatum* L.) Peel Extract as a Natural Source of Antioxidants

**DOI:** 10.3390/nano11061439

**Published:** 2021-05-29

**Authors:** Maral Soltanzadeh, Seyed Hadi Peighambardoust, Babak Ghanbarzadeh, Maryam Mohammadi, José M. Lorenzo

**Affiliations:** 1 Department of Food Science, College of Agriculture, University of Tabriz, Tabriz 5166616471, Iran; maral.soltanzadeh@gmail.com (M.S.); peighambardoust@tabrizu.ac.ir (S.H.P.); ghanbarzadeh@tabrizu.ac.ir (B.G.); ma.mohammadi@tabrizu.ac.ir (M.M.); 2 Drug Applied Research Center and Student Research Committee, Tabriz University of Medical Sciences, Tabriz 5165665811, Iran; 3 Centro Tecnológico de la Carne de Galicia, Rúa Galicia N°4, Parque Tecnológico de Galicia, San Cibrao das Viñas, 32900 Ourense, Spain; 4 Área de Tecnología de los Alimentos, Facultad de Ciencias de Ourense, Universidad de Vigo, 32004 Ourense, Spain

**Keywords:** pomegranate peel, nanocarrier, particles, nanostructure, bioactive compounds, properties

## Abstract

The encapsulation of pomegranate peel extract (PPE) in chitosan nanoparticles (CSNPs) is an advantageous strategy to protect sensitive constituents of the extract. This study was aimed to develop PPE-loaded CSNPs and characterize their physical, structural morphology, antioxidant and antimicrobial properties. Spherical NPs were successfully synthesized with a mean diameter of 174–898 nm, a zeta potential (ZP) of +3 – +36 mV, an encapsulation efficiency (EE) of 26–70%, and a loading capacity (LC) of 14–21% depending on their loaded extract concentrations. Based on these results, CSNPs with chitosan:PPE ratio of 1:0.50 (*w*/*w*) exhibited good physical stability (ZP = 27 mV), the highest loading (LC = 20%) and desirable encapsulation efficiency (EE = 51%), and thus, selected as optimally loaded NPs. The FTIR analysis of PPE-CSNPs demonstrated no spectral changes indicating no possible chemical interaction between the PPE and CSNPs, which confirms that the PPE was physically entrapped within NPs. Moreover, FTIR spectra of pure PPE showed specific absorption bands (at 3293–3450 cm^−1^) attributed to the incidence of phenolic compounds, such as tannic acid, ellagic acid and gallic acid. Total phenolic content (TPC) and antioxidant analysis of selected CSNPs revealed that the encapsulated NPs had significantly lower TPC and antioxidant activity than those of pure PPE, indicating that CSNPs successfully preserved PPE from rapid release during the measurements. Antibacterial tests indicated that pure PPE and PPE-loaded CSNPs effectively retarded the growth of Gram-positive *S. aureus* with a minimum inhibitory concentration (MIC) of 0.27 and 1.1 mg/mL, respectively. Whereas Gram-negative *E. coli,* due to its protective cell membrane, was not retarded by pure PPE and PPE-CSNPs at the MIC values tested in this study. Gas chromatography-mass spectroscopy analysis confirmed the incidence of various phytochemicals, including phenolic compounds, fatty acids, and furfurals, with possible antioxidant or antimicrobial properties. Overall, CSNPs can be regarded as suitable nanomaterials for the protection and controlled delivery of natural antioxidants/antimicrobials, such as PPE in food packaging applications.

## 1. Introduction

The food industry produces copious amounts of agricultural waste with potential application as the substrate for the recovery of added-value compounds [1,2]. In the recent era, there is a growing demand towards the use of herbal extracts, particularly those originated from plant byproducts, with potent use in food preservation [3], agricultural [4], pharmaceutical [5] and cosmetics [6]. The peels of pomegranate (*Punica granatum* L.) comprise roughly half of the fruit’s weight and are not directly consumed and discarded as waste. The peels are rich in bioactive compounds, such as polyphenols, flavonoids, proanthocyanidins, and hydrolyzable tannins compared to pomegranate juice and seeds [7,8,9,10]. It is reported that pomegranate peels possess strong antioxidant [11], antimicrobial [5,12], antiviral [13], anti-inflammatory [14], antimutagenic [15], and anticarcinogenic activities [16] and could be used for preservation and therapeutic applications. The antioxidant activity of pomegranate pericarp is due to its polyphenolic constituents (mostly punicalagin and ellagic acid), which, even at small concentrations, can inhibit oxidation processes and thus provide positive effects in the human body [8].

Most natural bioactive compounds and bio-preservatives are sensitive to oxidative reactions and, therefore, can be easily decayed by extreme environmental or processing conditions (e.g., oxygen, light, high temperatures, humidity, pH changes, etc.) [17,18]. This restricts their application compared to synthetic preservatives [19]. Encapsulation of bioactive materials into polymeric envelopes or reservoirs by producing nanosized particles [20,21] could compensate for this limitation. The advantages of encapsulating bioactive compounds in nanoparticles are: (i) the protection from adverse environmental effects, and thus, the extension of the shelf life of unstable compounds [22]; (ii) developing targeted-delivery, controlled- and effective-release nanomaterials to achieve a prolonged therapeutic and functional effects [23,24]; (iii) the improvement of physical characteristics and easier handling of the core bioactive materials [25]; and (iv) the covering of pungent odors/smells of some herbal extracts or essential oils and enhancing their food applicability and sensory acceptance [26].

Chitosan is a linear polysaccharide with cationic nature and high potential to encapsulate natural ingredients. This would result in developing different forms of chitosan matrix (nanoparticles, nanoemulsions, nanofibers, hydrogels, films and coatings). These systems can be used for the encapsulation of medicinal herbal extracts and essential oils for potential applications in the food industry, pharmaceutical and cosmetics [27]. Chitosan owing to its general recognition as safe (GRAS), has some advantages, such as non-toxicity, biocompatibility, and antimicrobial properties [23], which makes it suitable for in vivo use in biomedical treatments [28]. Chitosan nanoparticles (CSNPs) are easily fabricated by the ionic gelation method between the cationic linear chitosan polymer and anionic tripolyphosphate [29]. The CSNPs, due to their higher surface-to-volume ratio, provides the advantage of carrying natural extracts, which reinforce the functionality and compatibility of the nanoparticles [26]. From a technological point of view, nanoparticles also serve as strengthening fillers in the polymeric matrix by enhancing their barrier properties. Moreover, nanomaterial fillers can be regarded as suitable carriers for antimicrobial and antioxidant agents in preserving food quality [30,31,32,33]. Like other nanoparticles, CSNPs can be considered potential nano-reinforcing carrier material in many biopolymeric applications in food packaging.

Many reports describe the encapsulation of various herbal extracts in CSNPs and their potential applications [34]. The encapsulation of plant extracts, such as garlic [35], cherry [36], green tea [37], tea polyphenols [38], cranberry [39], *Arrabidaea chica* extract [40], flavonoids [41], phenolic compounds [42], and *Tridax procumbens* leaf extract [43] in CSNPs has been outlined. However, to the best of our knowledge, there is no study on different characteristics of CSNPs containing the extract of pomegranate peels. Therefore, the objective of this research was to prepare PPE-loaded chitosan nanoparticles and to investigate their physical, morphological, microstructural, antioxidant and antibacterial properties.

## 2. Materials and Methods

### 2.1. Chemicals

Low molecular weight (LMW) chitosan (Mw 55–180 kDa) with deacetylation degree of 74–84%, acetic acid, sodium tripolyphosphate (STPP), Folin–Ciocâlteu phenol reagent, DPPH (2,2-diphenylpicrylhydrazyl), methanol, ethanol, sodium carbonate, and Mueller-Hinton (MH) broth and MH agar media were prepared from Merck (Darmstadt, Germany). *Escherichia coli* (PTCC 1163) and *Staphylococcus aureus* (PTCC 25,923) as stock cultures were obtained from Persian Type Culture Collection (PTCC, Tehran, Iran).

### 2.2. Preparation of Pomegranate Peel Extract (PPE)

Fully ripened pomegranates (*Punica granatum* L.) of Rabab-e-Neiriz cultivar (Neiriz, Iran), characterized by thick peels [44], were freshly obtained in one batch from a local market. The fruits were skinned manually, and the peels were dried under shade at ambient temperatures for one week. The peels were pulverized and sieved through a 700 µm mesh. An amount of 500 g peel powder was soaked in a 2.5 L hydromethanol solution (with methanol: water ratio of 4:1) for 3 days at ambient temperature in the dark. The suspension was then shaken gently and filtered to separate the solids. The methanol was recovered from the remaining extract using a vacuum rotary evaporator (Heidolph Instruments Co., model VV 2000, Schwabach, Germany) at 40 °C. The obtained extract was then allowed to dry out to a moisture content of 20% at ambient temperature (23 °C) in the dark for 5 days and stored at −18 °C until use [45].

### 2.3. Preparation of Chitosan Nanoparticles (CSNPs) Loaded with PPE

CSNPs were prepared by ionic gelation method using STPP as a crosslinking agent. LMW chitosan (0.2 g) was added to 40 mL acetic acid (1% *v*/*v*) to achieve a 0.5% *w*/*v* concentration, and it was then kept overnight under magnetic stirring at ambient temperature to get a clear solution, and its pH was adjusted to 4.6 using a 1 M NaOH. Chitosan solution was then filtered through a 0.45 µm syringe filter. PPE at different loading contents was added to the CS solution according to formulations shown in Table 1.

Chitosan solution containing PPE was magnetically stirred (at 500 rpm) for 60 min at ambient temperature (23 °C). A stock solution of STPP was prepared in double-distilled water to achieve a concentration of 0.2% (*w*/*v*); it was then filtered through a 0.22 µm syringe filter, its pH was adjusted to 5.6 by 1 M HCL, and it was stored at 4 °C before use. Chitosan nanoparticles were spontaneously produced by gradually adding 10 mL of cold STPP solution using a syringe pump (with a flow rate of 0.25 mL/min) into 10 mL of chitosan solution containing PPE under vigorous stirring (1400 rpm) for 50 min while holding in an ice-bath. The obtained suspension was then centrifugated at 9000 rpm for 30 min [29]. The supernatant was taken for subsequent analysis, and sedimented CSNPs were collected as pellets and washed several times by deionized water. The wet pellets were then subjected to probe sonication (VCX 130, Vibra Cell Sonics, Newtown, CT, USA) at 130 W/20 kHz/80% amplitude for 20 min (sonication/resting cycles of 1 min) in an ice-bath to get homogenous suspensions. CSNPs were then lyophilized at −60 °C for 72 h and kept refrigerated for further analysis.

### 2.4. Characterization of the CNPs

#### 2.4.1. Total Phenolic Content (TPC)

TPC was determined using the Folin–Ciocâlteu (Fol–Ci) method [46] with the following modifications. An aliquot of 125 μL from Fol–Ci reagent was mixed with a 300 μL sample and 1825 μL distilled water followed by shaking the mixture for 5 min. Afterward, 250 μL of sodium carbonate solution (20% *w*/*v*) was added and shaken for another 5 min followed by standing for 30 min in a water bath at 40 °C. The absorbance of the samples was determined with a UV-vis spectrophotometer (Varian Cary 500, Agilent Technologies, Santa Clara, CA, USA) at 765 nm. Standard curves were prepared using GA (0–250 μg/mL), and the results were stated as mg GA equivalence (GAE)/g dry matter [47]. The amount of PPE was estimated by a suitable calibration curve of absorbance plots of pure PPE against different concentrations using Equation (1);

(1) $$Y=0.105x+0.005,R^{2}=0.99$$

#### 2.4.2. Mean Particle Size, Zeta Potential, and Polydispersity Index

The mean diameter (MD) of nanoparticles, zeta potential (ZP), and polydispersity index (PDI) of freshly prepared CSNPs with and without PPE loading were studied using dynamic light scattering (DLS) (Zetasizer-ZS, Malvern, UK) [48,49]. The ZP values of nanoparticles show the impact of core material loading on nanoparticles’ surface charge, which indicates NPs stability. The polydispersity index (PDI) indicates the overall uniformity of particles in suspension and can also show nanoparticle aggregation behavior [50].

#### 2.4.3. Scanning Electron Microscopy Observations

To observe the surface morphology of CSNPs, the samples were dried on glass slides and mounted on aluminum stubs, followed by gold thin layer coating using vacuum ion sputtering device (E−1010, Hitachi, Japan). Then, nanoparticles were observed using scanning electron microscopy (SEM) (MIRA3, TESCAN, Brno, Czech Republic) [51].

#### 2.4.4. Encapsulation Efficiency and Loading Capacity

The encapsulation efficiency (EE) is defined as the real content of material loaded into the NPs. A predetermined amount of freshly prepared PPE-loaded CSNPs was centrifuged at 9000 rpm for 35 min at 25 °C. The supernatant was analyzed for PPE using UV-vis spectrophotometry, as mentioned in Section 2.4.1. To determine the EE, the initial amount of PPE, which was equal to its loaded content inside NPs and free PPE (unloaded PPE in supernatant) was determined. The EE and loading capacity (LC%) were then calculated using Equations (2) and (3), respectively [52].

(2) $$\mathrm{EE}=\frac{\mathrm{Initial}\;\mathrm{amount}\;\mathrm{of}\;\mathrm{PPE}-\mathrm{Free}\;\mathrm{PPE}}{\mathrm{Initial}\;\mathrm{amount}\;\mathrm{of}\;\mathrm{PPE}}\times 100$$

(3) $$\mathrm{LC}=\frac{\mathrm{Initial}\;\mathrm{amount}\;\mathrm{of}\;\mathrm{PPE}-\mathrm{Free}\;\mathrm{PPE}}{\mathrm{Mass}\;\mathrm{of}\;\mathrm{carrier}\;\left(\mathrm{CSNPs}\right)}\times 100$$

#### 2.4.5. Fourier-Transform Infrared (FTIR) Spectroscopy

To study the potential interactions between chemical structural elements of the components used in the preparation of loaded CSNPs, Fourier-transform infrared (FTIR) spectra were obtained from 4000–400 cm^−1^ (FTIR spectrophotometer, Bruker-Tensor 27, Bremen, Germany) according to the method described previously [53].

#### 2.4.6. Antioxidant Activity

DPPH (2,2-diphenyl-1-picrylhydrazyl) free radical scavenging activity (RSA) of PPE and PPE-loaded CSNPs was determined according to the method reported by Shetta et al. [52] with some modifications as follows: PPE or PPE-loaded CSNPs were either dissolved or dispersed in ethanol to give a concentration of 1 mg/mL. It worth mentioning that PPE was used at a concentration equal to that of loaded in CSNPs at a CS: PPE ratio of 1:0.50. A 0.1 mL aliquot of diluted samples was mixed with a 3.9 mL ethanolic DPPH stock solution (0.1 mM) under vigorous mixing. The obtained solution was kept in the dark for 30 min, and the absorbance of the sample was measured at 517 nm using UV-vis spectrophotometry (Varian Cary 500, Agilent Technologies, Santa Clara, CA, USA) against the absorbance of the DPPH. DPPH-RSA (%) was calculated using Equation (4).

(4) $$\mathrm{DPPH}\;\mathrm{RSA}\;\left(\%\right)=\left[\frac{A_{\mathrm{Blank}}-A_{\mathrm{Sample}}}{A_{\mathrm{Blank}}}\right]\times 100$$

where *A*_Blank_ and *A*_Sample_ are the absorbances of the control and the sample.

#### 2.4.7. Antibacterial Activity

Antibacterial activity of PPE before and after loading in CSNPs was evaluated against Gram-negative *Escherichia coli* and Gram-positive *Staphylococcus aureus* using minimum inhibitory concentration (MIC) and minimum bactericidal concentration (MBC) tests [54,55]. The MIC was determined in a 96-well microtiter plate (Labsystems, Helsinki, Finland) using resazurin aided microdilution method in Mueller-Hinton (MH) broth as reported by Elshikh et al. [56] with some modifications as follow. Bacterial inoculation was prepared with concentrations adjusted to 1.5×10^8^ CFU/mL of McFarland turbidity standard at 0.5. The standardized suspensions were then further diluted by 1:100 in MH broth and kept incubated at 37 °C for 24 h. Microtiter plates were prepared according to stages 1–4, as shown in Table 2. 

The reduction of blue resazurin to pink resorufin indicates the viability of microbial cells because of their reductase enzyme action. By lasting the incubation time (4 h), the column with blue/purple color (no color change) indicated no microbial growth and thereby scored as MIC value. The MBC is considered as the minimum concentration of the material, which kills 99.9% of bacterial inoculum [52] by assaying the live microorganisms in those wells from the MIC test that showed no microbial growth (no color change).

#### 2.4.8. Gas Chromatography (GC)–Mass Spectrometer Analysis

Measurement of PPE components was done using a GC (GC-17A, Shimadzu Inc., Kyoto, Japan) connected to a mass spectrometer (Model QP-5050A) equipped with a DB-5 column (polydimethylsiloxane, dimensions: 60 m × 0.25 mm) (J&W Scientific, Folsom, CA, USA) and a flame-ionized detector, which was operated in the EI (electron ionization) mode at 70 eV. Helium as the carrier gas was used, and the flow rate was adjusted to 1.2 mL/min. The oven temperature was increased from 50 to 240 °C by a 3 °C/min rate. Injector and detector temperatures were set to 230 and 270 °C, respectively. The split ratio was adjusted to 1:10, and the injected volume was 1 μL of 10% PPE in n-hexane. The components were identified by comparing their mass spectra with reference chemicals available in the literature using computer matching with NIST107 and WILEY229 libraries [57].

### 2.5. Statistical Analysis

Statistical Analysis System (SAS) software (SAS version 9.2, SAS Inc., Cary, NC, USA) was used to establish Pearson’s correlations between the independent variable (PPE concentration levels, PPE-CL) and dependent variables (MD, ZP, PDI, EE, and LC). For dependent variables, such as SEM particles’ MD, TPC, DPPH, and MBC values, a one-way analysis of variance (ANOVA) was performed. Tukey’s multiple range test was used to determine the significant (*p* < 0.05) difference between the means.

## 3. Results and Discussion

### 3.1. Particle Size Distribution, Average Diameter, Zeta Potential, and Poly Disparity Index

**Figure 1:**
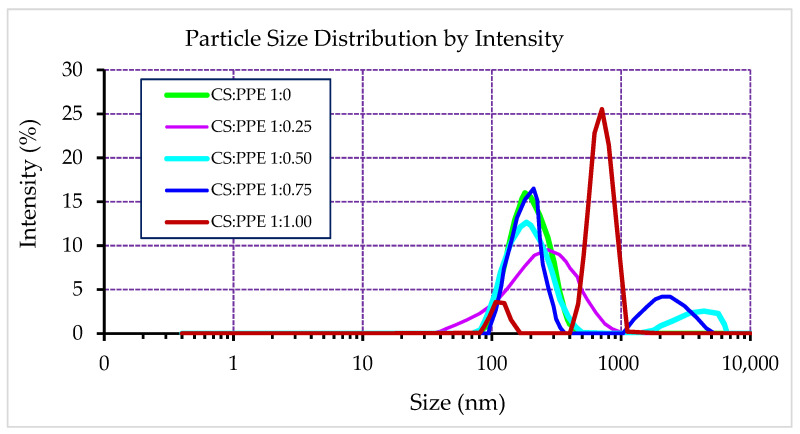
Intensity-based particle size distribution of CSNPs at different chitosan:PPE weight ratios.

The result of the size distribution measurement of PPE-loaded CSNPs at different chitosan:PPE weight ratios are shown in Figure 1.

This figure shows the intensity-weighted particle size distribution, which can be sensitive to small numbers of aggregated particles since the intensity is related to 10^6^-fold of particles radius. Thus, it can provide useful information about the existence of large particles or aggregates in the NPs system. As can be seen from this figure, empty CSNPs exhibited a narrow size distribution showing a low extent of polydispersity. CSNPs at lower PPE concentrations (chitosan:PPE ratios of 1:0.25 and 1:0.50) also showed unimodal size distribution. However, higher PPE loading concentrations shifted the size distribution curves to larger particle size domains showing a bimodal distribution with higher polydispersity. For example, PPE-loaded CSNPs with chitosan:PPE ratios of 1:0.75 and 1:1.00 exhibited large numbers of aggregated particles. This may be attributed to a possible leakage of PPE to the surface of these NPs, which could cause sticking and aggregation of NPs. Table 3 shows numerical values of particle size analysis by Malvern Zetasizer. Empty CSNPs showed the lowest mean particle size (Z-average diameter, 173.9 nm). By loading PPE, the mean particle size of NPs was significantly (*p* < 0.05) increased from 198 nm in chitosan:PPE ratio of 1:0.25 to 898 nm in chitosan:PPE ratio of 1:1.00. The zeta potential (ZP) of the nanoparticles indicates their surface charge and, thereby, their stability. As can be seen in Table 3, empty CSNPs showed significantly higher ZP values than that all PPE-loaded CSNPs. The higher ZP of empty CSNPs confirms forming spontaneous and stable nanocomplex between chitosan and STPP.

Incorporating PPE into these nanoparticles decreased their ZP values, indicating the weakening of their physical stability. Increasing PPE concentration significantly (*p* < 0.05) decreased ZP values from 26.5 mV in chitosan:PPE ratio of 1:0.50 to 2.95 mV in chitosan:PPE ratio of 1:1.00. There was no significant difference in ZP values of CSNPs with loading ratios of 1:0.25 and 1:0.50. A similar range of zeta potential was found in other studies, where CSNPs were developed and loaded with other herbal extracts [35,36,37,42] or essential oils [20,22,50,56]. As mentioned, the surface electrical charge of nanoparticles is an indicator of their stability. Based on Derjaguin–Landau–Verwey–Overbeek (DLVO) theory [58], there is a balance between attractive “van der Waals” and repulsive “electrostatic” forces at the surface of stable nanoparticles because of their higher positive surface charge. Thus, it can be assumed that higher surface charges can lead to the strong electrostatic repellent forces of nanoparticles, preventing them from aggregation. It also has been reported that ZP values > ±30 mV correspond to high stability, ZP of around ±20 mV indicates a moderate or short-term stability, and the ZP values around ±5 mV would result in low stability, and thereby a fast aggregation of NPs [52]. The lowest polydispersity index (PDI) is an indicator of the overall uniformity of nanoparticles. Higher PDI values indicate larger or aggregated particles. Lower PDIs correspond to monodispersed and small particles with no aggregation [50]. As can be seen from Table 3, empty CSNPs showed the lowest PDI values (0.25) among all NPs, indicating forming uniform and low-dispersed particles. Loading PPE in CSNPs increased their PDIs. By increasing PPE concentration, the PDI values were significantly (*p* < 0.05) increased from 0.260 in chitosan:PPE ratio of 1:0.25 to 0.682 in chitosan:PPE ratio of 1:1.00.

### 3.2. Pearson’s Correlation Between the Independent Variable (PPE Concentration Levels, CL) and Dependent Variables

**Figure 2:**
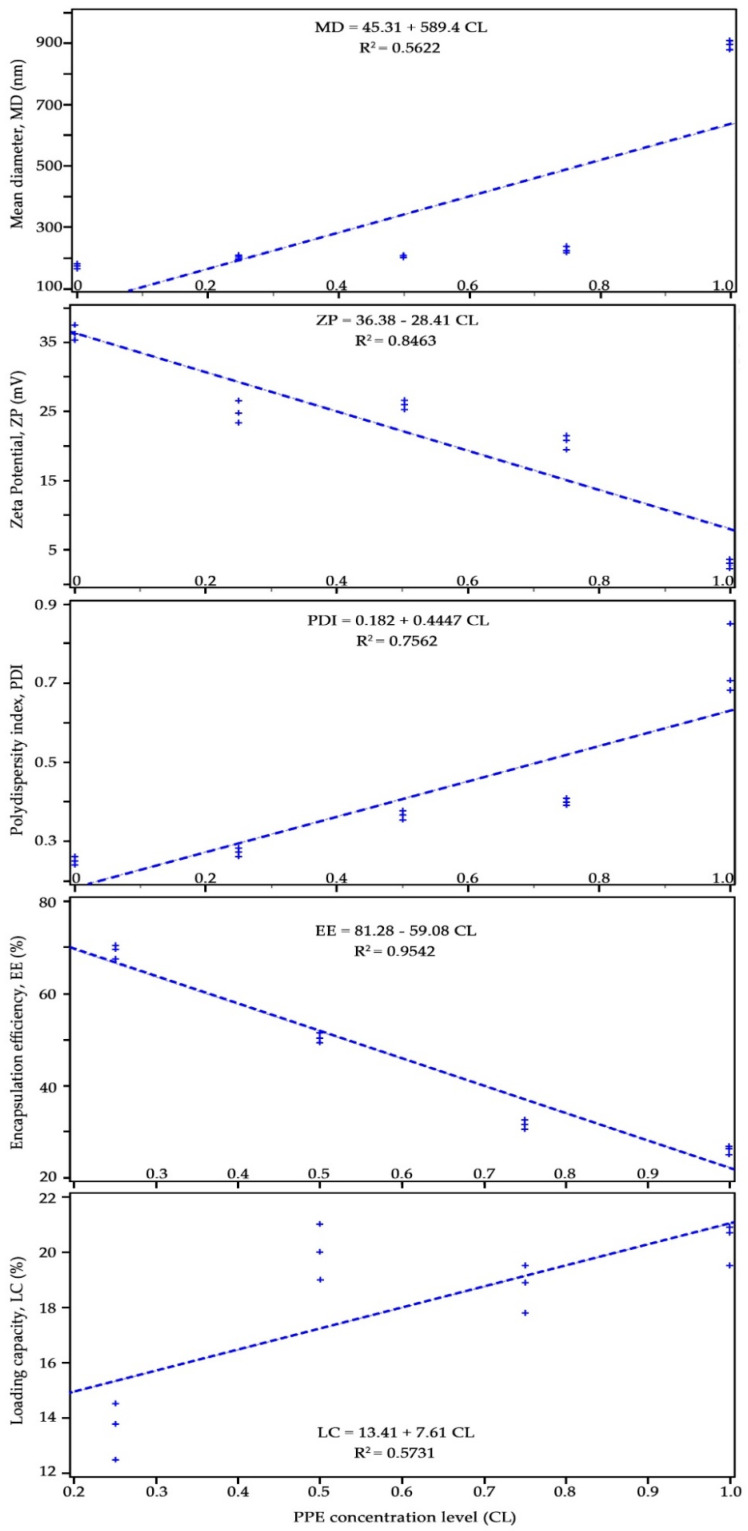
Pearson’s correlation between PPE concentration levels (as an independent variable) and dependent variables: MD: mean diameter; ZP: zeta potential; PDI: poly-dispersity index; EE: encapsulation efficiency; LC: loading capacity.

Effect of PPE concentration at five levels (chitosan:PPE ratio of 1:0, 1:0.25, 1:0.5, 1:0.75, and 1:1) on variations of NPs mean diameter (MD), zeta potential (ZP), poly-dispersity index (PDI), encapsulation efficiency (EE), and loading capacity (LC) was evaluated by constructing a Pearson’s correlation. The results are shown in Figure 2. As can be seen from this figure, dependent variables EE, ZP, and MD were most significantly (*p* < 0.001) correlated with the independent variable (CL) with *R*^2^ values of 0.95, 0.85, and 0.76, respectively. LC and MD variables with *R*^2^ values of 0.57 and 0.56, respectively showed less significant correlations at *p* = 0.013 and *p* = 0.0044, respectively compared to those of aforementioned variables. These results indicate that encapsulation efficiency and zeta potential are highly and negatively dependent on PPE concentration in chitosan nanoparticles. The polydispersity index and the particles’ mean diameter positively depended on the concentration level and showed increased values with increasing concentration of loaded PPE in chitosan nanoparticles. Scatter plot matrix data of all independent variables showed a similar significant (*p* < 0.001) correlation with concentration levels (data are not shown).

### 3.3. Scanning Electron Microscopy Observations

**Figure 3:**
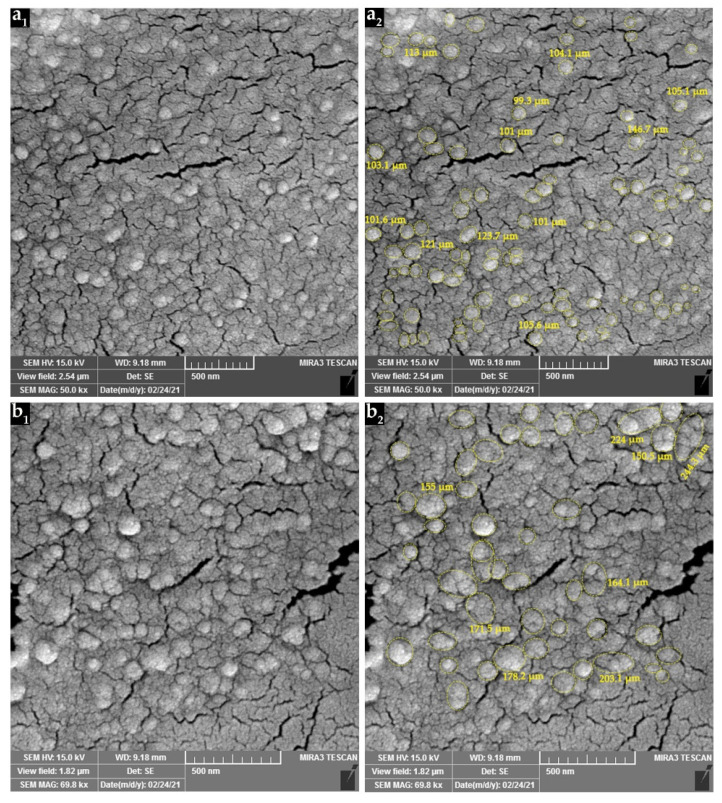
SEM image of (**a_1_**) empty CSNPs, and (**b_1_**) PPE-loaded CSNPs at chitosan:PPE ratio of 1:0.50 (*w*/*w*). Images (**a_2_**,**b_2_**) represent processed figures using ImageJ.

SEM was used to analyze the surface morphology and size distribution of CSNPs and PPE-loaded CSNPs (in chitosan:PPE ratio of 1:5.00 *w*/*w*). The results are shown in Figure 3a_1_,b_1_. SEM micrographs showed spherical morphology and homogenous size distribution in the case of both CSNPs. ImageJ (an open-source imaging freeware developed by the National Institutes of Health, MD, USA) was used to quantify particle size diameter and distribution [59,60]. After setting the scale of SEM images to the measured scale-bar value (500 nm), a manual mode of measuring particle diameter (length) was applied on the particles specified in Figure 3a_2_,b_2_ [61,62].

The results of particle number count, minimum, maximum and mean diameters, and standard deviations extracted from SEM images are shown in Table 4. The average size of the empty CSNPs was smaller than that of PPE-loaded CSNPs. SEM observations confirmed the results of dynamic particle size measurement obtained by Malvern Zetasizer (Table 3), and it was indicated that loading PPE into CSNPs increases mean particle size diameter. In addition, comparing size results obtained from SEM images with those measured by Malvern Zetasizer revealed that the size of NPs appears to be smaller in the SEM images.

### 3.4. Encapsulation Efficiency (EE) and Loading Capacity (LC)

The results of EE and LC are tabulated in Table 3. From UV-vis spectrophotometry, the amount of encapsulated PPE inside CSNPs as defined as EE was in the range of 26.3–69.7%. CSNPs with chitosan:PPE ratio of 1:0.25 exhibited a maximum amount of EE (69.7%), followed by CSNPs with chitosan:PPE ratio of 1:0.50 (50.5%). From Table 3, it can be seen that CSNPs with chitosan:PPE ratio of 1:0.50 (*w*/*w*) provides the highest ZP value indicating good physical stability with a high loading capacity and the second-best encapsulation efficiency, and thereby it was selected for the rest of the analysis. Further increase of PPE in CSNPs decreased EE values. The decrease of EE for the sample prepared using higher concentrations of PPE may be explained by the encapsulation limitation in these NPs. In addition, the LC of PPE-loaded CSNPs was in the range of 13.8–20.7% (Table 3). The LC increased by increasing the concentration of PPE from 1.25 to 5% *w*/*v*. This finding was in agreement with other studies, where LC values were increased as a function of initial drug content [63,64].

### 3.5. FTIR Spectroscopy

**Figure 4:**
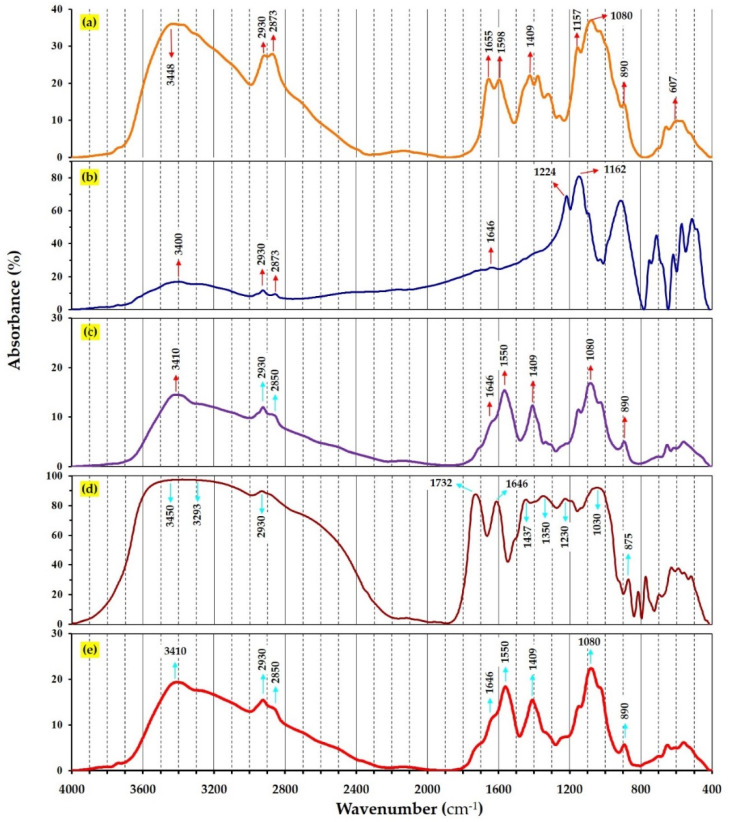
FT-IR spectra of (**a**) chitosan, (**b**) STPP, (**c**) empty CSNPs, (**d**) PPE and (**e**) PPE-loaded CSNPs (at chitosan:PPE ratio of 1:0.50 *w*/*w*).

Fourier-transform infrared (FTIR) technique was used to characterize the chemical structure of components involved in pomegranate extract loaded CSNPs. FTIR spectra of CS, STPP, PPE, CSNPs and PPE-loaded CSNPs are shown in Figure 4, which reveals the occurrence of multiple functional groups in their structure. CS powder (Figure 4a) showed characteristic peaks at 3448 (for stretching vibration of OH and NH_2_), 2930 (C–H bond vibrations in alkanes), 1655 (C=O bond vibrations in the amide I molecules), 1373 (general OH groups bending), 1157 and 1080 (correspond to the stretching vibrations of C–O–C bonds), and 607 cm^−1^ (correspond to the vibration of pyranoside rings), which are also confirmed by Shetta et al. [52]. It is expected that crosslinking of CS polymer with STPP molecules would shift the peaks related to amide groups. Thus, comparing FTIR spectra of chitosan, STPP and CSNPs in Figure 4a–c reveals that the peak at 3448 cm^−1^ (–NH_2_ groups stretching vibration of in CS) was shifted to 3410 cm^−1^ in CSNPs attributing to the occurrence of STPP molecules [65]. In addition, the peaks at 1655 and 1598 cm^−1^ in CS (relating to C=O stretching of the amide I) were shifted to 1646 and 1550 cm^−1^ in CSNPs, indicating that the amine groups of CS and polyanionic phosphate groups of STPP may take part in the reaction [65]. Furthermore, the strong band at 1168 cm^−1^ related to –COOH groups of TTP was not seen in the FTIR spectrum of CSNPs, indicating that chitosan was completely crosslinked with STPP [15].

As can be seen in Figure 4d, absorption bands of PPE functional groups are defined. The broadband between 3293 and 3450 cm^−1^ is ascribed to stretching vibration of N–H and O–H bands, which can be possibly found in tannic acid, ellagic acid and gallic acid, which has been confirmed in other studies [15,66]. The peaks at 2930, 1732, and 1646 cm^−1^ were Attributed to C–H, C=O, and carbonyl groups. The relatively small bands that appeared at 1437, 1350 and 1230 cm^−1^ are assigned to vibrations of the aromatic ring in PPE. The strong band at 1030 cm^−1^ ascribed to C–O stretching of carboxylic acid. FTIR analysis was used to evaluate whether the encapsulation nature of PPE within CSNPs is chemical or physical entrapment. If no or minimal changes of FTIR spectrum were observed than the parental compounds, one could expect a physical entrapment, whereas spectral shift would be attributed to possible chemical interaction between PPE and CSNPs. Comparing FTIR spectra of CSNPs (Figure 4c) and PPE-loaded CSNPs (Figure 4e) exhibited no spectral changes confirming that PPE was physically entrapped (encapsulated) within CSNPs. In addition, adding PPE to CSNPs resulted in a significant increase in the intensity of C–H stretching bands at 3410, 2930, 2850, 1550, 1409, and 1080, reflecting successful incorporation of PPE into CSNPs. Our results were in agreement with those reported in earlier studies [23,52].

### 3.6. Total Phenolic Content

**Figure 5:**
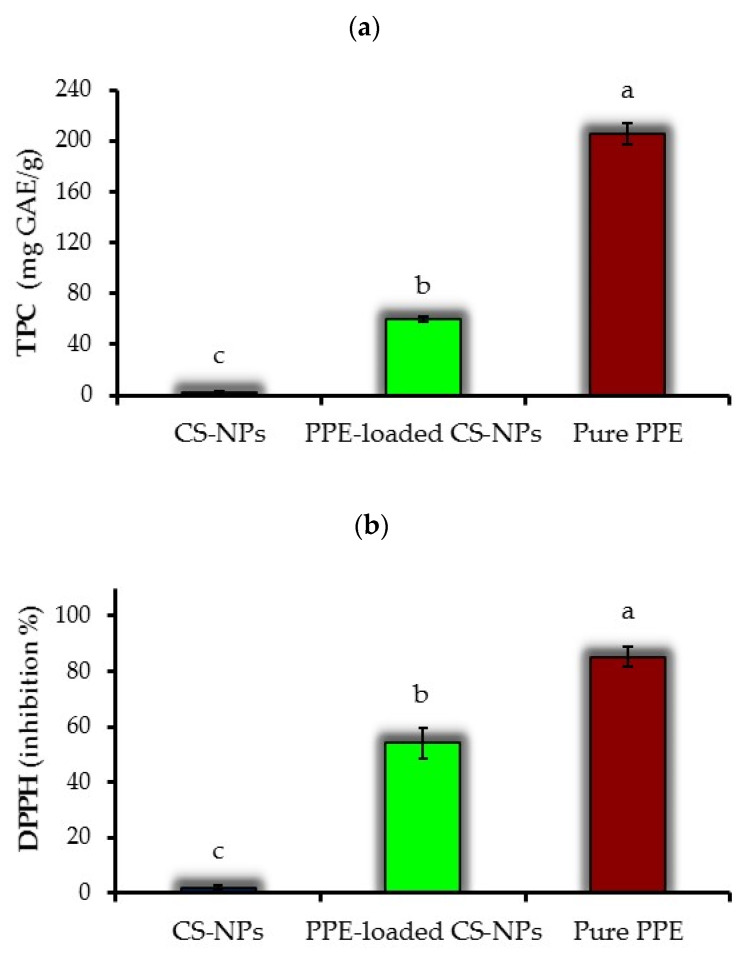
Results of (**a**) TPC and (**b**) DPPH-RSA measured for PPE, empty CSNPs and PPE-loaded CSNPs (at chitosan:PPE ratio of 1:0.50 *w*/*w*). Data represent the average of triplicate measurements. Error bars show standard deviations. Different alphabetical letters indicate significant (*p* < 0.05) differences between means.

The results of TPC determination for pure PPE, CSNPs and PPE-loaded CSNPs (at chitosan:PPE ratio of 1:0.50) are presented in Figure 5a. Based on these results, each gram of CSNPs, PPE-loaded CSNPs and pure PPE is equivalent to 2.5, 59.6 and 205.2 mg GAE. Despite the non-phenolic nature of CSNPs, they indicated some traces of total phenolic content, which may be attributed to the chromogen compounds formed during Fo–Ci reagent reaction with non-phenolic reducing substances. Moreover, the TPC of PPE-loaded CSNPs was significantly (*p* < 0.05) lower than that of pure PPE. The amount of pure PPE used in TPC analysis was similar to its concentration used to prepare PPE-loaded CSNPs (at chitosan:PPE ratio of 1:0.50 *w*/*w*). Thus, the lower TPC value of CSNPs compared to pure PPE shows a good encapsulation efficiency of these NPs that preserve PPE from leaching out during the measurement. However, based on previous studies [23,52], it can be expected that extended storage of NPs may lead to increased release of their core material. Esmaeili and Asgari [67] also indicated that CSNPs incorporating *Carum copticum* essential oil preserve encapsulated bioactive material during TPC assay.

### 3.7. DPPH Radical Scavenging Activity

Figure 5b shows DPPH RSA results measured for PPE-loaded CSNPs, free PPE, empty CSNPs. The antioxidant activity of PPE-loaded CSNPs (56% inhibition) was significantly lower than that of pure PPE (85% inhibition). The amount of pure PPE used in the DPPH assay was similar to PPE-loaded CSNPs (at chitosan:PPE ratio of 1:0.50 *w*/*w*). Thus, lower DPPH value of CSNPs than pure PPE might be due to the protective effect of CSNPs in restricting the rapid leakage of PPE from chitosan nano-capsules during the DPPH assay, as also was seen for TPC data (Figure 5a). Empty CSNPs showed a limited antioxidant activity (1.9% inhibition), which could be related to crosslinking between chitosan poly-cation and STPP poly-anion [68]. Similar results for the limited antioxidant activity of CSNPs were reported by Shetta et al. [52] and Chen et al. [69].

### 3.8. Antimicrobial Activity

**Figure 6:**
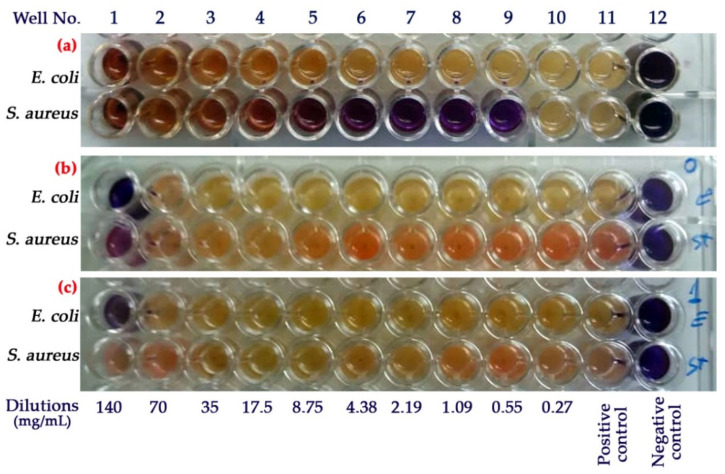
Results of antimicrobial test against *E. coli* and *S. aureus* using resazurin-aided microdilution method performed in 96-well microtiter plates. Samples: (**a**) PPE, (**b**) empty CSNPs, (**c**) PPE-loaded CSNPs (chitosan:PPE ratio of 1:5.00 *w*/*w*). Sample concentrations for well columns 1–10 are reported in the figure.

In previous studies, the antibacterial properties of pomegranate extract have been outlined [15]. However, it seems that components originated from different parts of fruit would exhibit various antimicrobial activities [12]. Bioactive compounds of PPE, such as tannins, polyphenols, punicalagin and ellagic, have been reported to possess antibacterial activity [7,68]. The results of antibacterial activity based on MIC test for PPE, CSNPs and PPE-loaded CSNPs against *E. coli* and *S. aureus* are shown (Figure 6a–c). First of all, positive controls (MH broth + Bacteria) showed distinct color changes from blue (for resazurin) to pink/yellowish (for resorufin), confirming good cell viability and growth of both *S. aureus* and *E. coli*. In contrast, the negative control (only broth) showed no color change representing the sterility of the MH broth medium. As can also be seen in Figure 6a, free PPE exhibited no antibacterial activity against *E. coli*, while MIC and MBC values of 0.27 and 0.55 mg·mL^−1^ were obtained for *S. aureus*, respectively (Table 5). 

It has to be mentioned that pure PPE was used at a concentration equal to that of loaded in CSNPs at a CS:PPE ratio of 1:0.50. No antibacterial effect of PPE against *E. coli* may be because Gram-negative bacteria have a multilayer complex membrane structure consisting of phospholipids, lipopolysaccharide, and a rigid exoskeleton peptidoglycan layer protecting the cell against antimicrobials [69]. Such membrane structure is not seen in Gram-positive bacteria (i.e., *S. aureus*), making them susceptible to cell lysis.

According to Figure 6b, empty CSNPs did not show antibacterial properties against both bacteria. A possible explanation for this effect may be the involvement of chitosan in the crosslinked STPP molecules limiting the release of cationic chitosan within NPs. This can be supported by results in Figure 5a,b, where it was demonstrated that empty CSNPs do not exhibit biological activity in terms of TPC and DPPH RSA activity.

PPE-loaded CSNPs, as compared to pure PPE, showed lower antibacterial activity in terms of MIC and MBC values against *S. aureus* (Figure 6c and Table 5). This may indicate that the encapsulation of PPE inside CSNPs successfully limits the leakage of PPE from NPs during the time interval between the preparation and antimicrobial assay.

### 3.9. GS-MS Analysis of the PPE

**Figure 7:**
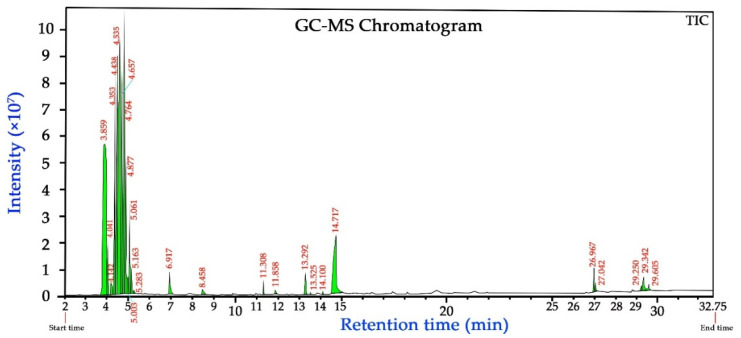
GC–MS chromatogram of methanolic extract of pomegranate peel.

Analysis of methanolic extract of pomegranate peel using GC–MS showed various phytochemical components representing 100% of the total extract composition. The peaks in the chromatogram (Figure 7) were identified qualitatively based on their retention times and mass spectral patterns.

Table 6 represents the identified components of the GC-chromatogram based on different retention times and mass spectral patterns using the NIST107 and WILEY229 Mass Spectral Library database of known compounds stored in the GC–MS library. A total of 28 compounds were detected, out of which the major compounds identified as hexane (42.2%), methanol (18.09%), 2,3-dimethylpentane (10.13%), heptane (6.64%), hydroxymethyl furfurol (HMF) (6.05%), methyl-cyclopentane (5.33%), cyclohexane (3.89%), and ethanol (1.57%). The 9,12-octadecadienoic acid and n-hexadecanoic acid are characteristic secondary metabolites found in many plants have been shown to possess several biological properties, such as antimicrobial and anti-inflammatory hypocholesterolemic, cancer preventive, hepatoprotective, and antioxidants [70].

## 4. Conclusions

Pomegranate peel extract (PPE) was successfully encapsulated within chitosan nanoparticles (CSNPs) using the ionic gelation technique. It was shown that the loading concentration PPE affects the physical characteristics of the obtained NPs. The optimal concentration of PPE was obtained at a chitosan:PPE ratio of 1:0.50 (*w*/*w*). The resulting NPs exhibited spherical morphology, unimodal and narrow size distribution with an average particle diameter of 208 nm, and possessed desirable encapsulation efficiency (EE) and positive surface charge (zeta potential), indicating their physical stability. However, increasing the loading concentration of PPE increased the average particle size, polydispersity and exhibited large particle aggregates. The loading capacity (LC) and EE were increased and decreased, respectively, as a function of PPE loading concentration. FTIR results indicated no spectral change between the components of PPE-CSNPs, confirming physical entrapment or encapsulation of PPE within CSNPs. PPE-loaded CSNPs showed significantly lower TPC and DPPH RSA values compared to those of pure PPE, confirming a successful encapsulation of PPE within CSNPs. MIC and MBC antibacterial test revealed the antibacterial activity of PPE against *S. aureus*. Overall, CSNPs provide an effective nanomaterial to encapsulate and protect the sensitive extract of pomegranate peels.

## Figures and Tables

**Table 1:** Different formulations (treatments) to prepare chitosan nanoparticles (CSNPs) incorporating pomegranate peel extract (PPE).

Treatments	LMW−CS (mL)(solids, mg)	PPE (mL)(solids, mg)	TPP (mL)(solids, mg)	Total (mL)(solids, mg)
CS:PPE of 1:0	10.0 (50)	0	10 (20)	20 (70.00)
CS:PPE of 1:0.25	9.0 (45)	1.0 (11.25)	10 (20)	20 (76.25)
CS:PPE of 1:0.50	9.0 (45)	1.0 (22.50)	10 (20)	20 (87.50)
CS:PPE of 1:0.75	9.0 (45)	1.0 (33.75)	10 (20)	20 (98.75)
CS:PPE of 1:1.00	9.0 (45)	1.0 (45.00)	10 (20)	20 (110.0)

**Table 2:** Preparation steps of microtiter plate for the MIC test.

**Wells (column no.) →**		1	2	3	4	5	6	7	8	9	10	11	12
**Stages**	**Concentrations (%)**	100	50	25	12.5	6.25	3.13	1.56	0.78	0.39	0.19	Positive control (Broth + Bacteria)	Negative control (Broth)
**1**	**Addition of** **MH broth (µL)**	-	100	100	100	100	100	100	100	100	100	100	100
**2**	**Addition of** **extract or NPs (µL)**	100	100	Serial two-fold dilutions for wells 3–10								0	0
**3**	**Addition of** **Bacteria (µL)**	100	100	100	100	100	100	100	100	100	100	100	0
**4**	**Addition of resazurin (µL)**	30	30	30	30	30	30	30	30	30	30	30	30

**Table 3:** Effect of PPE loading at different concentrations on average diameter, zeta potential (ZP), and poly-dispersity index (PDI) of CSNPs.

Chitosan:PPE (*w*/*w*)	Z-average Diameter (nm)	Zeta Potential (mV)	Poly-Dispersity Index (PDI)	Encapsulation Efficiency (EE%)	Loading Capacity (LC%)
1:0	173.9 ± 3.6 *^e^	36.3 ± 0.85 ^a^	0.250 ± 0.011 ^d^	-	-
1:0.25	198.0 ± 4.1 ^d^	24.8 ± 0.89 ^b^	0.260 ± 0.015 ^d^	69.7 ± 1.05 ^a^	13.8 ± 0.15 ^c^
1:0.50	208.2 ± 4.8 ^c^	26.5 ± 1.80 ^b^	0.368 ± 0.012 ^c^	50.5 ± 1.25 ^b^	20.0 ± 0.38 ^a^
1:0.75	224.1 ± 5.8 ^b^	20.8 ± 0.85 ^c^	0.399 ± 0.013 ^b^	31.7 ± 0.95 ^c^	18.9 ± 0.21 ^b^
1:1.00	897.7 ± 35.4 ^a^	2.95 ± 0.22 ^d^	0.682 ± 0.035 ^a^	26.3 ± 1.55 ^d^	20.7 ± 0.57 ^a^

* Data are mean of triplicate measurements ± SD. Different alphabetical letters in each column show significant (*p* < 0.05) differences between means.

**Table 4:** Numerical values of particle size extracted from SEM images using ImageJ processing.

Chitosan:PPE (*w*/*w*)	n	Particle Diameter or Length (nm)			
		Average	St. Dev	Min	Max
Empty CSNPs	100	90.6	21.5	51.5	148.0
PPE-loaded CSNPs	46	127.3	38.7	66.9	244.3

**Table 5:** MIC and MBC results of pure PPE and PPE-loaded CSNPs (at chitosan:PPE ratio of 1:0.50 *w*/*w*) against *E. coli* and *S. aureus*.

**Microbial strain→**	*E. coli*		*S. aureus*	
**Samples**	MIC (mg/mL)	MBC (mg/mL)	MIC (mg/mL)	MBC (mg/mL)
**PPE**	-	-	0.27	0.55
**PPE-loaded CS-NPs**	-	-	1.09	2.19

**Table 6:** GC–MS characterization of the phytochemical components of pomegranate (*Punica granatum* L., Rabab-e-Neiriz cultivar) peel extract.

Peak No.	RT (min)	Percentage	Identified Compounds	Molecular Weight (Da)	Molecular Formula
1	3.701	0.05	Unknown	-	-
2	3.859	18.09	Methanol	32	CH_4_O
3	4.041	1.57	Ethanol	46	C_2_H_6_O
4	4.142	0.43	Glycidol	74	C_3_H_6_O_2_
5	4.189	0.84	Glycolamide; 2-hydroxy-acetamide	75	C_2_H_5_NO_2_
6	4.353	6.64	Heptane	100	C_7_H_16_
7	4.438	10.13	2,3-Dimethyl pentane	100	C_7_H_16_
8	4.535	20.57	n-Hexane	86	C_6_H_14_
9	4.657	8.96	Hexane	86	C_6_H_14_
10	4.764	12.67	1-Hexene	84	C_6_H_12_
11	4.877	4.71	Methyl-cyclopentane	84	C_6_H_12_
12	5.003	0.62	Methyl-cyclopentane	84	C_6_H_12_
13	5.061	2.84	Cyclohexane	84	C_6_H_12_
14	5.163	0.92	Cyclohexane	84	C_6_H_12_
15	5.283	0.13	Cyclohexane	84	C_6_H_12_
16	6.917	0.92	Furfural (furan derivatives)	96	C_5_H_4_O_2_
17	8.458	0.29	2,5-Furandione; 3-methyl-citraconic anhydride	112	C_5_H_4_O_3_
18	11.305	0.32	1,8-Cineole; terpene; eucalyptol; p-cineole	154	C_10_H_18_O
19	11.860	0.14	Furancarboxylic acid; furoic acid; methyl furate	126	C_6_H_6_O_3_
20	13.296	0.78	2,3-Dihydro-,3,5-dihydroxy-4H-pyran-4-one,	144	C_6_H_8_O_4_
21	13.522	0.07	Camphor; 1,7,7-trimethyl-bicyclo[2.2.1] heptan-2-one	152	C_10_H_16_O
22	14.103	0.07	Borneol; 1,7,7-trimethyl-(1s endo)-bicyclo[2.2.1]heptan-2-one	154	C_10_H_18_O
23	14.720	6.05	5-Hydroxymethyl-2-furancarboxaldehyde; hydroxymethyl furfurole (HMF)	126	C_6_H_6_O_3_
24	26.968	0.73	2-Methoxy-phenol; guaiacol	124	C_7_H_8_O_2_
25	27.039	0.29	n-Hexadecanoic acid; palmitic acid	256	C_16_H_32_O_2_
26	29.249	0.23	(z,z)-9,12-Octadecadiennoic acid; linoleic acid	280	C_18_H_32_O_2_
27	29.399	0.64	(z)-9-Octadecenoic acid; oleic acid	282	C_18_H_34_O_2_
28	29.605	0.3	n-Octadecanoic acid; stearic acid	284	C_18_H_36_O_2_
Total	-	100%	-	-	-

## Data Availability

The date presented in this study are available in the article.
